# Efficacy of letrozole versus clomiphene citrate on ovulation induction in patients with polycystic ovarian syndrome

**DOI:** 10.12669/pjms.38.5.5565

**Published:** 2022

**Authors:** Mehnaz Khakwani, Rashida Parveen, Shazia Yousaf, Ayesha Uzaima Tareen

**Affiliations:** 1 Mehnaz Khakwani, FCPS, Department of Obstetrics and Gynecology, Unit-1, Nishtar Medical University, Multan, Pakistan; 2 Rashida Parveen, FCP, Department of Obstetrics and Gynecology, Unit-1, Nishtar Medical University, Multan, Pakistan; 3 Shazia Yousaf, FCPS, Department of Obstetrics and Gynecology, Unit-1, Nishtar Medical University, Multan, Pakistan; 4 Ayesha Uzaima Tareen, FCPS, Department of Obstetrics and Gynecology, Unit-1, Nishtar Medical University, Multan, Pakistan

**Keywords:** Polycystic ovarian syndrome, Letrozole, Clomiphene citrate

## Abstract

**Objectives:**

To compare the efficacy of letrozole (LTZ) vs clomiphene citrate (CC) for ovulation induction in patients having polycystic ovarian syndrome (PCOS).

**Methods:**

This randomized controlled trial was conducted at The Department of Obstetrics & Gynecology, Nishtar Medical University Hospital, Multan, Pakistan from January 2021 to June 2021. A total of 78 women aged 18 to 30 years, diagnosed having PCOS were enrolled. In Group-A, 39 women were given LTZ, 5mg for five days of menstrual cycle. In Group-B, 39 women were given CC, 100mg for five days of menstrual cycle. All patients underwent transvaginal scan (TVS) for the evaluation of efficacy in terms of ovulation induction.

**Results:**

Overall mean age was noted to be 25.41±2.84 years. Most of the patients, 51 (65.4%) belonged to rural area of residence. There were 52 (66.7%) patients with BMI less than 25 kg/m^2^. Overall, mean duration of infertility was found to be 2.62±0.74 years. Among 70 patients who completed the follow ups and analyzed regarding efficacy, in Group-A, efficacy was noted in 23 (59.0%) patients in comparison to 14 (35.9%) in Group-B (p=0.0413). Mean endometrial thickness was significantly better in Group-A versus Group-B (8.1±1.5 mm vs. 6.8±1.9 mm, p=0.0022).

**Conclusion:**

Aiming ovulation induction, letrozole in comparison to clomiphene citrate was found to have significantly better efficacy among women having anovulatory PCOS.

## INTRODUCTION

Polycystic ovarian syndrome (PCOS) is known to be a frequent endocrine disorder and considered to a common cause of infertility, depressive symptoms and life dissatisfaction.^1^ PCOS is estimated to affect 20 to 33% of women of reproductive age while 50% of the women with PCOS are obese.^2^ Local data shows infertility because of ovulatory dysfunction to be around 21% among women of reproductive age.^3^,^4^ During PCOS, ovaries secrete relatively more androgens and halt ovulation. Irregular menstruation, hyperandrogenism and infertility are the presenting problems in women with PCOS.^5^ Obesity is the most common feature of PCOS that further aggravates insulin resistance.^6^

Various strategies are adopted for the induction of ovulation like weight reduction, metformin and laparascopic ovarian drilling. The most popular treatment options for infertility among with PCOS is through medications like gonadotrophins, pituitary follicular stimulating hormone therapies and aromatase inhibitors.^7^,^8^ Variation exists between induction of ovulation and pregnancy rates between different treatment options considered in PCOS.^9^

Letrozole (LTZ) is an aromatase inhibitor and in comparison to CC, LTZ is linked with better thickening of endometrium and pregnancy rates while congenital anomalies are comparable between the two drugs.^8^ Begum MR and Colleagues comparing rates of induction of ovulation between LTZ and CC found LTZ to result in successful induction of ovulation rates as 63.6% in comparison to 29.4% with the CC.^10^ Badawy A et al did not find any significant difference between LTZ and CC in terms of induction of ovulation rates (67.5% versus 70.9%, p>0.05).^11^ In Pakistan, not much work is seen comparing the induction of ovulation rates in women with PCOS treated with LTZ versus CC while published literature shows variations.

The objective of this study was to compare the efficacy of LTZ vs CC for ovulation induction in women having PCOS.

## METHODS

This randomized controlled trial was conducted in the “Department of Obstetrics & Gynecology, Nishtar Medical University Hospital, Multan, Pakistan” from January 2021 to June 2021 after the approval from “Institutional Ethical Committee” (Ref#23483/NMU&H, dated:03-12-2020). Informed consent was sought from participants of this study. This trial was registered at www.ClinicalTrials.gov as “NCT05075863”. Sample size of 78 (39 in each group) women was estimated considering 95% confidence interval, 80% power of test and presumed efficacy of LTZ and CC as 63.3% and 29.4% respectively.^10^

A total of 78 women aged 18 to 30 years with PCOS presenting in outpatient department (OPD) of Obstetrics & Gynecology, Nishtar University Hospital, Multan” were included. Diagnosis of PCOS was made as existence of any two of these: peripherally arranged 2-9 follicles of 8-10mm, increased LH and FSH ratio above two, history of oligomenorrhea (less than 6 cycles / years) or serum testosterone above 0.65 ng/ml.

### Exclusion criteria:

Women with thyroid disorders as shown in TSH between 0.5 to 5 IU/L, T3 between 4.3 to 8.6 Pmol/l and T4 between 9 to 22 mol/l or those with hyperprolactinemia (serum prolactin above 400 miu/l) were excluded. Women having duration of infertility above five years, or infertility in the partner, malignancy involving the breast or ovary were also no included.

**Fig.1:**
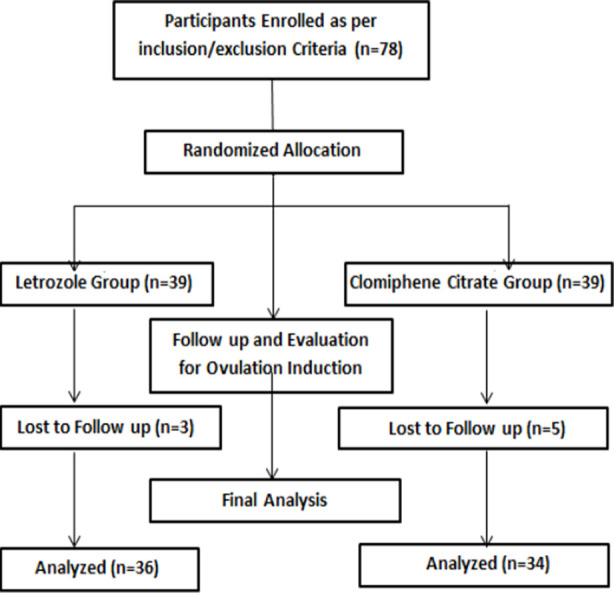
Methodology flow chart.

Detailed demographic history including name, age, body mass index (BMI), duration of infertility and area of residence were recorded. All women were allocated randomly to two groups through computer generated numbers (Fig.1). Women in Group-A were given LTZ, 5mg from day three to seven of menstrual cycle. In Group-B, 39 women were given CC, 100mg from 3 to 7 days of menstrual cycle. All women underwent transvaginal scan (TVS) for the evaluation of ovulation induction. If follicle of more than 2 cm was located on 12^th^ day on TVS and/or small/collapsed on 16^th^ day on TVS, ovulation induction was considered yes or otherwise. Endometrial thickness was also evaluated. A special proforma was designed to record all study data.

SPSS version 26.0 was used for data analysis. Qualitative data like area of residence and efficacy were presented by frequency and percentages. The quantitative data like age, duration of infertility, BMI and endometrial thickness were presented as mean±SD. Post stratification chi-square was applied. Independent sample t-test was used to compare endometrial thickness. P value < 0.05 was considered significant.

## RESULTS

Overall mean age was noted to be 25.41±2.84 years while 70 (89.7%) patients were aged between 21 to 30 years. Most of the patients, 51 (65.4%) belonged to rural area of residence. Overall, mean BMI (kg/m^2^) was noted to be 23.74±2.96 while there were 52 (66.7%) patients with BMI less than 25 kg/m^2^. Overall, mean duration of infertility was found to be 2.62±0.74 years. Comparison of characteristics of study participants in between both study groups are shown in Table-I. No statistical difference was found (p>0.05).

**Table I:** Characteristics of Study Participants (n=78).

Characteristics		Group-A (n=39)	Group-B (n=39)	P-Value
Age	≤20	5 (12.8%)	3 (7.7%)	0.4554
	21-30	34 (87.2%)	36 (92.3%)	
Area of Residence	Urban	12 (30.8%)	15 (38.5%)	0.4752
	Rural	27 (69.2%)	24 (61.5%)	
BMI (kg/m^2^)	<25	24 (61.5%)	28 (71.8%)	0.3367
	≥25	15 (38.5%)	11 (28.2%)	
Duration of Infertility (years)	<3	30 (76.9%)	27 (69.2%)	0.4438
	≥3	9 (23.1%)	12 (30.8%)	

There were eight women who were lost to follow up (3 in Group-A and 5 in Group-B) so 70 patients were included in the final analysis (36 in Group-A and 34 in Group-B). Distribution of efficacy among patients of both study groups is shown in Table-II. Overall, efficacy was noted in 37 (47.4%) women. In Group-A, efficacy was noted in 23 (59.0%) patients in comparison to 14 (35.9%) in Group-B (p=0.0413).

**Table II:** Distribution of Efficacy (n= 70).

Efficacy	Group-A (n=36)	Group-B (n=34)	P-Value
Yes	22 (61.1%)	12 (35.3%)	0.0308
No	14 (38.9%)	22 (64.7%)	

Mean endometrial thickness was noted to be 8.1±1.5 mm in Group-A in comparison to 6.8±1.9 mm in Group-B while the difference turned to be statistically significant (p=0.0022).

## DISCUSSION

For some decades, clomiphene citrate has been considered as the 1^st^ choice of treatment for women having PCOS. CC is known to reduce uterine receptivity which could be a reason that it results in reducing the likelihoods of conception. Few of the major drawbacks of clomiphene citrate include poor efficacy rates (around 22% of livebirths), high chances of multiple pregnancy (3-8%).^12^,^13^ Some of the most frequent side-effects with CC include mood changes and hot flushes.^14^

In the present study, it was noted that following five days of treatment in both study groups, ovulation induction occurred on 14^th^ day of menstrual cycle in 35.9% patients who used CC in comparison to 59.0% patients in LTZ group (p=0.0413). A study evaluating use of CC up to a period of six months noted 25% failure of ovulation induction while live birth rate was 22.5%. It has been stated that CC results in ovulation inductions rates between 50-85% but the conception rates are not very high (15-27%).^15^-^17^ LTZ being an aromatase inhibitor is not found to influence adverse anti-estrogenic effects on endometrial receptivity while the documented ovulation as well as conception rates are higher.^18^,^19^ We noted significantly more patients to report ovulation induction in LTZ group when compared to patients using clomiphene citrate (59.0 vs 35.9%, p=0.0413). Ashfaq A et al in a previous local study found LTZ to result in 67.6% ovulation rates while CC resulted in ovulation induction in 32.4% of study participants.^20^ Begum MR et al also noted LTZ to result in significantly better rates of ovulation induction when compared to CC.^10^ Debashi S et al found LTZ to result in 60% ovulation induction rates in comparison to 32% with CC.^21^ Contrary to our results, Bayer U et al in a prospective randomized clinical trial found no significant difference in terms of ovulation induction between LTZ and CC groups (65.7% versus 74.7%, p=0.17).^22^ Difference in inclusion criteria as well as different study designs could be possible reasons behind these variations. LTZ has also been found to result in better efficacy in comparison to CC among patients undergoing assisted reproduction.^23^ Mean endometrial thickness was significantly better among women who were given LTZ Group-A versus Group-B (8.1±1.5 mm vs. 6.8±1.9 mm, p=0.0022). These results were in accordance to a study from Turkey where the authors noted that LTZ resulted in significantly improved endometrial thickness in comparison to CC (p<0.001).^8^

### Limitations:

As this was a single center study with a relatively small sample size, the results cannot be generalized. As we noted efficacy in both treatment groups as per defined criteria, we did not record antral follicle count which would have given us further insight about these drugs. We noted ovulation induction and we did not plan to analyze live-birth of pregnancy outcomes. More prospective trials having randomized nature and involving larger sample size will further confirm the findings of this study.

## CONCLUSION

Aiming ovulation induction, letrozole was found to have significantly better ovulatory efficacy in comparison to clomiphene citrate among women having PCOS.
